# Risk stratification of HPV 16 DNA methylation combined with E6 oncoprotein in cervical cancer screening: a 10-year prospective cohort study

**DOI:** 10.1186/s13148-020-00853-1

**Published:** 2020-05-07

**Authors:** Li Dong, Li Zhang, Shang-Ying Hu, Rui-Mei Feng, Xue-Lian Zhao, Qian Zhang, Qin-Jing Pan, Xun Zhang, You-Lin Qiao, Fang-Hui Zhao

**Affiliations:** 1 Department of Cancer Epidemiology, National Cancer Center/National Clinical Research Center for Cancer/Cancer Hospital, Chinese Academy of Medical Sciences and Peking Union Medical College, 17 South Panjiayuan Lane, P.O. Box 2258, Beijing, 100021 China; 2 Institutes of Biomedical Sciences, Shanxi University, Taiyuan, China

**Keywords:** Human papillomavirus, Viral methylation, E6 oncoprotein, Cervical cancer, Risk stratification

## Abstract

**Background:**

How to best triage human papillomavirus (HPV) positive women remains controversial in an era of HPV primary screening of cervical cancer. Here, we assessed the long-term risk stratification for triaging HPV 16 positive women by standalone HPV 16 methylation and combined with E6 oncoprotein.

**Methods:**

A total of 1742 women underwent screening with HPV DNA testing, cytology, and visual inspection with acetic acid (VIA) in 2005 and were followed for 10 years. Seventy-seven women with HPV 16 positivity determined by HPV genotyping test were examined via E6 oncoprotein detection and bisulfite pyrosequencing for quantitative methylation of L1 and LCR genes of HPV 16.

**Results:**

The 10-year cumulative incidence rate (CIR) of cervical intraepithelial neoplasia grade 3 or severe (CIN3+) for HPV 16 positive women was 25.3% (95% CI 14.7–37.3%), which significantly increased in women with high methylation at six sites (CpG 5602, 6650, 7034, 7461, 31, and 37) and in women with positive E6 oncoprotein. A methylation panel based on the above six sites showed a competitive risk stratification compared to cytology (HR 11.5 vs. 8.1), with a higher 10-year CIR of CIN3+ in panel positives (57.2% vs 36.8%) and comparable low risk in panel negatives (5.7% vs 4.8%).The sensitivity and specificity for accumulative CIN3+ was 85.7% (95%CI 60.1–96.0%) and 78.4% (95%CI 62.8–88.6%) for a methylation panel and 57.1% (95%CI 32.6–78.6%) and 86.5% (95%CI 72.0–94.1%) for E6 oncoprotein. The AUC values of methylation standalone and the co-testing of methylation panel and E6 oncoprotein were around 0.80, comparable to 0.68 for cytology, 0.65 for viral load, and superior to 0.52 for VIA (*p* < 0.05).

**Conclusions:**

Our findings indicated the promising use of HPV 16 methylation alone or combined with E6 oncoprotein for triaging HPV 16 positive women based on the long-term risk stratification ability.

## Introduction

Cervical cancer is the fourth most common female malignancy worldwide both in incidence and mortality, with an estimated 569,000 new cases and 313,365 new deaths in 2018 [1]. The worldwide consensus of persistent infections with high-risk human papillomavirus (hrHPV) as an essential etiology of cervical cancer is pushing forward an emerging era of HPV-based primary cervical cancer screening [2, 3]. The superior sensitivity of HPV testing to cytology has been demonstrated [4–6]. However, most HPV infections are transient, leading to its positivity prediction value and specificity far from ideal. There comes the challenge on how to best manage HPV positive women by differentiating those with high risk that would progress to precancers to reduce the excessive referral and overtreatment [7].

The cytology-based triaging strategy of HPV positive women, albeit ideal and effective, is a challenge for resource-limited areas [8], due to lack of trained cytopathologists, limited healthcare resources, and poor infrastructure. Therefore, an objective testing which isn’t resource-intensive would greatly facilitate the triage implementation. HPV genome methylation, a normal epigenetic event where functionally relevant changes to the genome are made without changing the nucleotide sequence, shows type-specific variation within the viral life cycle and differs during carcinogenesis. Abnormal DNA methylation may alter viral oncogene expression and thereby promote the carcinogenesis [9]. Mounting studies are supporting the promise of HPV DNA methylation as a triage tool of HPV positive women [10–12]. Genomic methylation HPV16, the most prevalent and carcinogenic HPV type for cervical cancer, is widely studied. Hypermethylation of the HPV16 L1, L2, E2, and E4 regions is associated with an increased risk of CIN3 and HPV persistent infections, and hypermethylation of the E6 gene is associated with a lower likelihood of high-grade cervical lesion [13–15]. Furthermore, the promising predictive property of the dynamic increase of HPV 16 methylation over time for persistent infections was also observed in samples collected at time points 0 to 7 years before CIN3 diagnosis [12]. However, the determination of the optimal hypermethylated CpG sites of HPV 16 to identify women at increased risk of cervical cancer remain controversial [13, 15–17].

More recently, Clarke et al. evaluated the clinical performance of a multi-type methylation assay composed of a total of 12 types of hrHPV genotype for detection of CIN3/AIS and indicated the apparent advantages of multi-type methylation assay as a triage method in terms of higher sensitivity and lower colposcopy referral [18]. Additionally, HPV DNA methylation assay is free from preservation of intact cells and able to potentially integrate into HPV DNA testing, which makes it to be one of the options for triging hrHPV-positive women in the Eurogin roadmap 2017 for cervical cancer screening[7].

Continuous E6 expression, inactivating the pro-apoptotic tumor suppressor p53, was crucial for the maintenance of the malignant phenotype and oncogenic transformation into the cervical cancer [19, 20]. Previous studies demonstrated that the detection of hrHPV E6 oncoprotein, i.e., the Onco***E6***™ Cervical Test, could serve as risk predictors of  high-grade cervical lesions [21, 22]. In our recent cross-sectional study, E6 oncoprotein showed a good “trade-off” between sensitivity and specificity in managing HPV-positive women [23]. However, the long-term risk triaging performance of E6 oncoprotein detection remains to be determined.

The association of HPV DNA methylation with E6 oncoprotein has never been verified in the population, albeit with molecular biology-based evidence [24]. Moreover, the clinical performances of their combinations in managing HPV positive women are underinvestigated. To address this gap, we analyzed the long-term risk stratification of HPV 16 DNA methylation patterns alone/in combination with E6 oncoprotein expression for incident cervical intraepithelial neoplasia grade 3 or severe (CIN3+) based on a 10-year follow-up cohort, with comparison of cytology, viral load, and visual inspection with acetic acid (VIA).

## Results

**Fig. 1:**
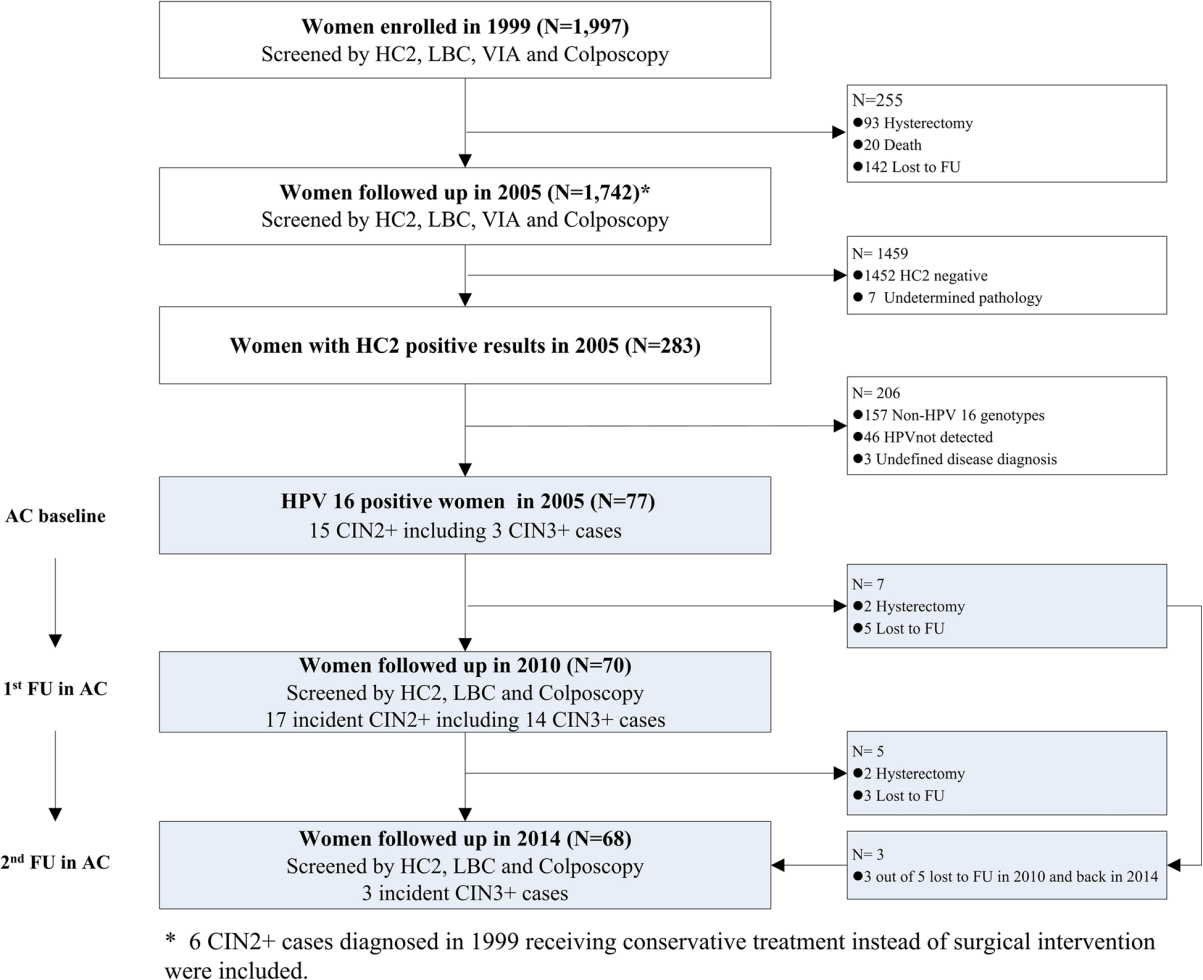
Flowchart of HPV 16 positive women follow-up in Shanxi Province Cervical Cancer Screening I (SPOCCS I) study, China, 1999–2014. *HC2* Hybrid Capture 2, *LBC* liquid-based cytology, *VIA* visual inspection with acetic acid, *CIN* cervical intraepithelial neoplasia, *CIN2+* CIN grade 2 or worse, *CIN3+* CIN grade 3 or worse, *AC* analytic cohort, *FU* follow up

A total of 1742 women were followed up in 2005 because 255 out of 1997 women recruited in 1999 were excluded due to either lost to follow-up, death, or hysterectomy during 1999–2005. Among those, 283 women were hrHPV positive including 77 HPV 16 positive women in 2005, the analytic cohort (AC), as shown in Fig. 1.

### High DNA methylation associated with increased cumulative risk of incident CIN3+

**Fig. 2:**
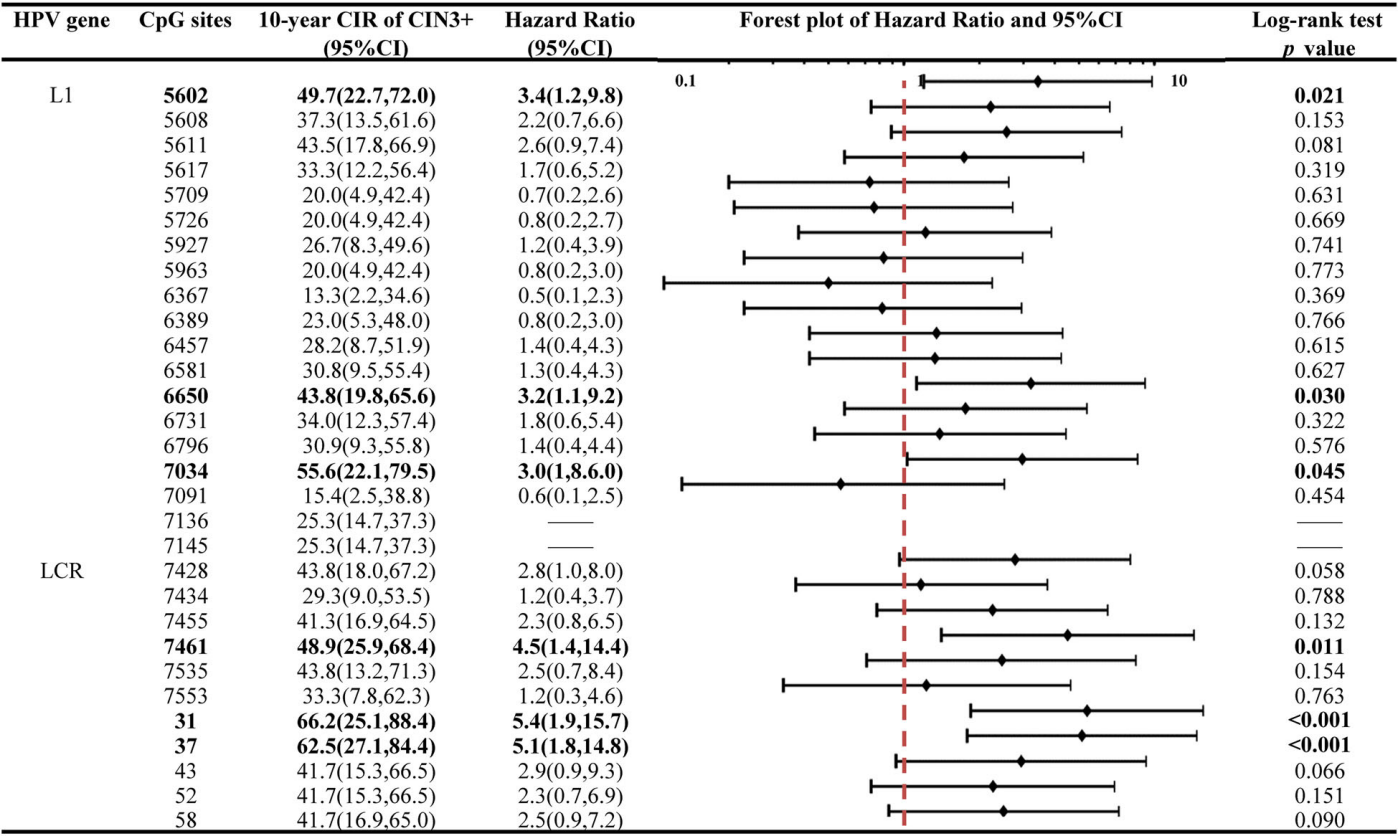
Ten-year cumulative incidence rate and hazard ratio of CIN3+ in HPV 16 positive women with site-specific high methylation of HPV 16. *HPV* human papillomavirus, *L1* Late gene 1, *LCR* long control region, *CIN3+* cervical intraepithelial neoplasia grade 3 or worse, *CIR* cumulative incidence rate

The DNA methylation levels at an individual CpG site of HPV 16 L1 and LCR gene ranged from 0 to 83.1% with higher median methylation levels in HPV16 L1 gene than those in LCR gene (18.5% vs 7.4%, *p* < 0.05). The same pattern was found even after stratification by histology-confirmed disease outcomes (Supplementary Figure 1). Ten-year cumulative risk of CIN3+ for different methylation statuses at each specific CpG site was evaluated (Fig. 2), with an overall 10-year CIR of CIN3+ of 25.3% (95% CI 14.7–37.3%) in HPV 16 positive women. High methylation at CpG 5602, 6650, 7034, 7461, 31, and 37 of HPV 16 were significantly associated with the high 10-year CIR of CIN3+, ranging from 43.8% (95% CI 19.8-65.6%) to 66.2% (95% CI 25.1-88.4%) with corresponding HR between 3.0 (95% CI 1.8–6.0) to 5.4 (95% CI 1.9–15.7) compared to the negative counterpart. The similar pattern with 10-year CIR of CIN3+ was observed for 5-year CIR of CIN3+ or 5-year or 10-year CIR of CIN2+ as disease outcomes (Supplementary Figures 2, 3 and 4).

Cumulative incident risks of CIN3+ among women with different numbers of high-methylation sites of HPV 16 L1 and LCR regions were evaluated, and higher risks were found in those with more numbers of high-methylation sites (Table 1). Women with more than two significant high-methylation sites of L1 and LCR regions of HPV 16 had a 12-fold (95% CI 2.5–57.7) increased risk compared to women with only one significant site. The similar patterns with 10-year CIR of CIN3+ were observed for 10-year CIR of CIN2+, 5-year CIR of CIN2+ and 5-year CIR of CIN3+ as endpoints (Supplementary Table 1). Meanwhile, we analyzed the methylation status of six significant CpG site in 2005 in all CIN3 and cancer cases and observed almost all six sites were high methylated in two cases of cancers (Supplementary Table 2).

**Table 1:** Cumulative incident risk of CIN3+ and HPV16 positivity frequency by the numbers of high-methylation sites of HPV 16

HPV gene	Numbers of sites*	Numbers of individual	10-year cumulative CIN3+			HPV16 positivity frequency^#^			
			Incidence rate	HR (95%CI)	*p*^§^	Once	Twice	Three times	*p*^**†**^
L1	0	26	7.7 **(**2.1, 24.1)	1 **(**Ref.)	< 0.001	76.9 **(**58.0, 89.0)	19.2 **(**8.5, 37.9)	3.8 **(**0.7, 18.9)	< 0.001
	1	28	21.4 **(**10.2, 39.5)	3.3 **(**0.7, 16.5)		40.0 **(**23.4, 59.3)	20.0 **(**8.9, 39.1)	40.0 **(**23.4, 59.3)	
	2–3	8	75.0 **(**40.9, 92.9)	14.6 **(**2.9, 74.6)		25.0 **(**7.1, 59.1)	0 **(**0, 32.4)	75.0 **(**40.9, 92.9)	
LCR	0	29	6.9 **(**1.9, 22.0)	1 **(**Ref.)	0.001	55.2 **(**37.6, 71.6)	20.7 **(**9.8, 38.4)	24.1 **(**12.2, 42.1)	0.296
	1	21	23.8 **(**10.6, 45.1)	3.9 **(**0.8, 20.2)		63.2 **(**41.0, 80.9)	10.5 **(**2.9, 31.4)	26.3 **(**11.8, 48.8)	
	2–3	12	58.3 **(**32.0, 80.7)	12.8 **(**2.6, 62.5)		36.4 **(**15.2, 64.6)	18.2 **(**5.1, 47.7)	45.5 **(**21.3, 72.0)	
L1+LCR	0	14	0 **(**0, 21.5)	---		71.4 **(**45.4, 88.3)	21.4 **(**7.6, 47.6)	7.1 **(**1.3, 31.5)	0.004
	1	22	9.1 **(**2.5, 27.8)	1 **(**Ref.)	< 0.001	59.1 **(**38.7, 76.7)	18.2 **(**7.3, 38.5)	22.7 **(**10.1, 43.4)	
	2	14	28.6 **(**11.7, 54.7)	3.7 **(**0.7, 20.0)		50.0 **(**25.4, 74.6)	16.7 **(**4.7, 44.8)	33.3 **(**13.8, 60.9)	
	3–6	12	66.7 **(**39.1,86.2)	12.0 **(**2.5,57.7)		27.3 **(**9.7,56.6)	9.1 **(**1.6,37.7)	63.6 **(**35.4,84.8)	

*HPV* human papillomavirus, *L1* Late gene 1, *LCR* long control region, *CIN3+* cervical intraepithelial neoplasia grade 3 or worse **(**CIN3+), *HR* hazard ratio, *CI* confidence interval

*Number of high methylation sites out of six significant sites of HPV 16: CpG 5602, 6650 and 7034 in L1 gene and 7461, 31 and 37 in LCR gene

^#^HPV genotyping test was conducted on all HC2 positive samples to determine HPV 16 positivity frequency in three visits of follow-up, i.e., in 2005, 2010, and 2014. Therefore, HPV16 positivity frequency may be once, twice, and three times in three tests during 2005–2014

^**§**^*p* value for log-rank test; ^**†**^*p* value for chi-square test

### High DNA methylation associated with HPV 16 E6 oncoprotein and HPV 16 positivity frequency

**Fig. 3:**
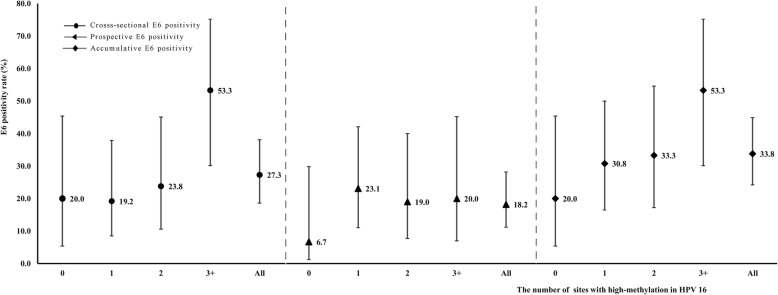
HPV 16 E6 positivity rates associated with the numbers of high-methylation sites of HPV 16. *HPV* human papillomavirus, *L1* Late gene 1, *LCR* long control region

The number of high-methylation sites in 2005 were positively related with the E6 oncoprotein positivity rates in 2005 (cross-sectional) and those in 2014 (prospective), and the positive relation still existed when using the 10-year cumulative E6 oncoprotein positivity rates (E6 positive in 2005 or 2014) as the outcome (Fig. 3). The 10-year cumulative E6 positivity rate remained about 20% among women with none high-methylation sites, which increased to approximated 30% among women with one or two high-methylation sites and 50% among those with at least three high-methylation sites.

The positive relation between the number of high-methylation sites at baseline and HPV16 positivity frequency during 10 years of follow-up was found, especially for the CpG sites in L1 gene (*p* < 0.001) (Table 1). The proportion of HPV 16 three-time positivity during 10 years of follow-up among women with no high-methylation sites was 7.1% (95% CI 1.3–31.5%), which was up to 22.7% (95% CI 10.1–43.4%), 33.3% (95% CI 13.8–60.9%), and 63.6% (95% CI 35.4–84.8%) among women with one, two, and at least three high-methylation sites, respectively.

### Long-term risk stratification for incident CIN3+ by standalone DNA methylation or combined with E6 oncoprotein

**Fig. 4:**
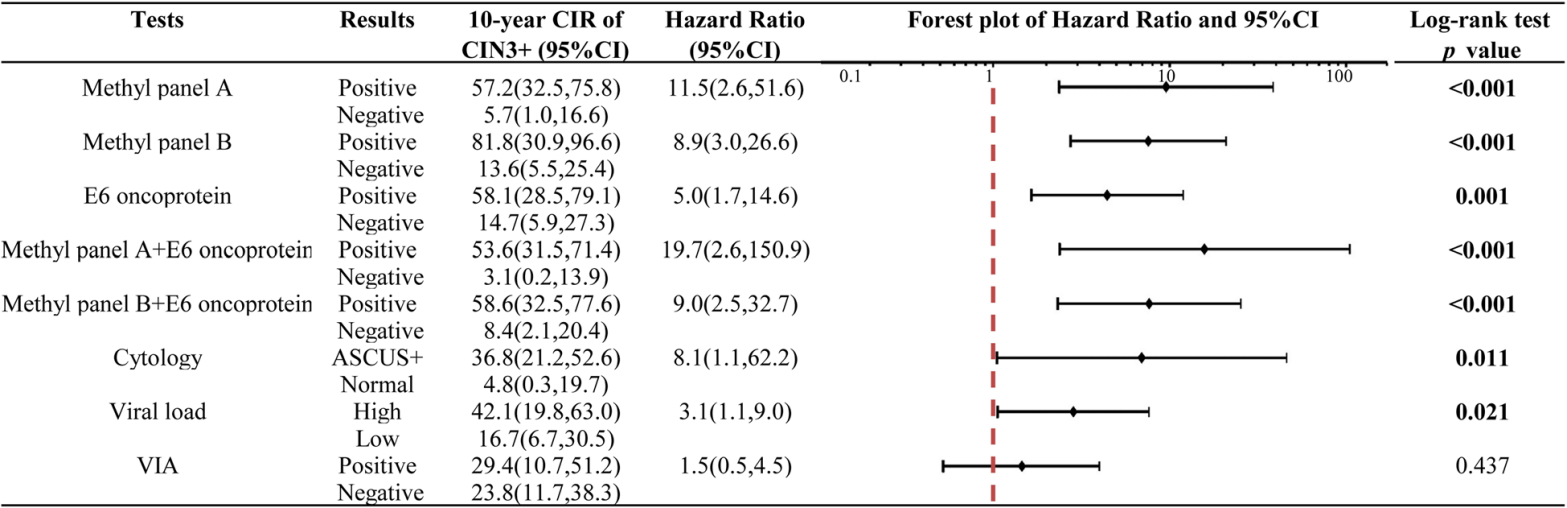
Cumulative incident risk of CIN3+ in HPV 16 positive women by triage methods. * HPV* human papillomavirus, *L1* Late gene 1, *LCR* long control region, *CIR* cumulative incidence rate, *CIN3+* cervical intraepithelial neoplasia grade 3 or worse, *VIA* visual inspection with acetic acid

As the number of significantly high-methylation sites indicated the 10-year accumulative risk of CIN3+, methylation panels combining them were explored. The positive panel A referred to at least two out of six sites (CpG 5602, 6650, 7034, 7461, 31, and 37) with high methylation, and at least three out of these six sites with high methylation for panel B. The 10-year CIRs of CIN3+ among women with positive panel A was 11.5 (95% CI 2.6–51.6) times higher compared to those with negative counterparts. Similarly, the hazard ratio (HR) for positive panel B reached 8.9 (95%CI 3.0–26.6) (Fig. 4). Women with positive E6 oncoprotein had five times higher 10-year CIR than those with negative E6 oncoprotein (95%CI 1.7–14.6). Combination with E6 oncoprotein, methylation panel A and panel B enhanced the 10-year CIRs to 19.7 and 9.0 folds, respectively. Cytology showed moderate risk stratification (HR 8.1, 95%CI 1.1–62.2), but viral load and VIA tended to have weaker risk stratification ability with HR of 3.1 (95%CI 1.1–9.0) and 1.5 (95%CI 0.5–4.5), respectively (Fig. 4).

### Triaging performance of standalone DNA methylation or combined with E6 oncoprotein

The triage performances of different methods for the 10-year cumulative CIN3+ were described in Table 2. Methylation panel B showed a highest specificity of 94.6% (95%CI 82.3-98.5%) and a decreased colposcopy referral rate to less than one of fifth. The specificity of methylation panel A was lower, but the sensitivity reached 85.7% (95% CI 60.1-96.0%). Methylation panels alone showed promise with competitive AUC values of 0.82 for panel A and 0.76 for panel B, compared to 0.72 for E6 oncoprotein, 0.68 for cytology, 0.65 for viral load and superior to 0.52 for VIA (*p* < 0.05). Combined with E6 oncoprotein, methylation panel A improved the sensitivity to 92.9% with reduced specificity of 73.0% but with the stable AUC value. Similar performance for methylation panel B was found. Of note, two incident cases of invasive cervical cancers detected during follow-up both showed positive panel A and B result at baseline.

**Table 2:** Clinical performance of HPV 16 methylation and E6 oncoprotein in triaging HPV 16 positive women for the detection of CIN3+

Triage strategies	Referral rate (%, 95% CI)	Sensitivity (%, 95% CI)	Specificity (%, 95% CI)	PPV (%, 95% CI)	NPV (%, 95% CI)	Youden index (%, 95% CI)	AUC (%, 95% CI)
Methyl panel A	39.2 (27.0, 52.9)	85.7 (60.1, 96.0)	78.4 (62.8, 88.6)	60.0 (38.7, 78.1)	93.5 (79.3, 98.2)	64.1 (52.2, 76.0)	0.82 (0.69, 0.91)
Methyl panel B	19.6 (11.2, 32.5)	57.1 (32.6, 78.6)	94.6 (82.3, 98.5)	80.0 (49.0, 94.3)	85.4 (71.6, 93.1)	51.7 (39.3, 64.1)	0.76 (0.62, 0.87)
E6 oncoprotein	25.5 (15.6, 38.9)	57.1 (32.6, 78.6)	86.5 (72.0, 94.1)	61.5 (35.5, 82.3)	84.2 (69.6, 92.6)	43.6 (31.3, 55.9)	0.72 (0.58, 0.84)
Methyl panel A+E6 oncoprotein	45.1 (32.3, 58.6)	92.9 (68.5, 98.7)	73.0 (57.0, 84.6)	56.5 (36.8, 74.4)	96.4 (82.3, 99.4)	65.9 (54.1, 77.7)	0.83 (0.70, 0.92)
Methyl panel B+E6 oncoprotein	35.3 (23.6, 49.0)	78.6 (52.4, 92.4)	81.1 (65.8, 90.5)	61.1 (38.6, 79.7)	90.9 (76.4, 96.9)	59.7 (47.5, 71.9)	0.80 (0.66, 0.90)
Cytology	66.7 (53.0, 78.0)	92.9 (68.5, 98.7)	43.2 (28.7, 59.1)	38.2 (23.9, 55.0)	94.1 (73.0, 99.0)	36.1 (24.1, 48.1)	0.68 (0.54, 0.80)
Viral load	35.3 (23.6, 49.0)	57.1 (32.6, 78.6)	73.0 (57.0, 84.6)	44.4 (24.6, 66.3)	81.8 (65.1, 91.4)	30.1 (18.7, 41.5)	0.65 (0.50, 0.78)
VIA	33.3 (22.0, 47.0)	35.7 (16.4, 61.2)	67.6 (51.5, 80.4)	29.4 (13.3, 53.1)	73.5 (56.9, 85.4)	3.3 (− 1.1, 7.7)	0.52 (0.37, 0.66)

*HPV* human papillomavirus, *CIN3+* cervical intraepithelial neoplasia grade 3 or worse, *Methyl*, methylation, *VIA* visual inspection with acetic acid, *PPV* positive predictive value, *NPV* negative predictive value, *CI* confidence interval, *AUC* area under the curve

## Discussion

This study is among the first to assess the predictive ability of HPV 16 methylation alone or in combination with E6 oncoprotein for incident CIN3+ compared to other triage methods based on a 10-year followed-up cervical cancer screening cohort. Our findings showed the 10-year cumulative risk stratification of CIN3+ in HPV 16 positive women by the use of dichotomized HPV 16 site-specific methylation and panel-specific methylation consisiting of six CpG sites (CpG 5602, 6650, 7034, 7461, 31, and 37). With the combination of E6 oncoprotein, methylation panels presented a competitive clinical performance in managing HPV 16 positive women with AUC values of 0.72–0.82, compared to cytology of 0.68 and viral load of 0.65, superior to VIA of 0.52. HPV16 DNA methylation was strongly associated with viral E6 oncoprotein expression and HPV infection frequency, suggesting series of active viral events both in epigenetics and gene translation in the formation of precancer and cervical cancer.

Previous studies indicated that HPV16 genome hypermethylation may be useful in predicting concurrent or even future development of cervical precancer and cancer [11, 12, 25]. Our finding provided additional evidence of the potential of elevated methylation levels of HPV 16 in predicting the future 10-year risk at the formation of precancers and cervical cancer. This finding was consistent with Mirabello et al.’s observations of significantly higher methylation levels at numerous CpG sites in HPV 16 L1 and LCR in pre-diagnostic three years of CIN2+ specimens compared to women with clearance of HPV 16 infection within 2 years [12]. Another recent study from the POBASCAM trial showed that DNA methylation can predict the future risk of cervical cancer over the subsequent 14 years [26]. Nevertheless we also noticed that HPV16 had many significantly hypermethylated CpG sites that presented strong associations with cervical carcinogenesis in cross-sectional studies [27], yet not all CpG sites sustained the hypermethylation status when viral infection persisted or progressed to cancer [12]. Methylation of HPV DNA may be a host response to foreign intracellular agents, a method of evading immune recognition; therefore, it might be a natural conservation for the viral to resist against any epigenetic changes of viral genome, which may partly explain the fact that only very few HPV CpG sites remained with pronounced predictive capacity.

With regard to specific CpG sites of HPV 16 with good predictive utility in our study, CpG 5602 in L1 gene had won the top AUC value in the detection of CIN3/AIS in Clarke et al.’s nested case-control study reaching 0.84 [18]; CpG 6650 and 7034 in L1 gene in two studies of Chinese population also showed strong association with high grade cervical lesions [17, 28]. However, methylation signatures in the top performing sites in Mazumder ID’s studies such as CpG 6367 and CpG 6389 did not present a good risk stratification effect for HPV 16 positive women in our current study [31]. Albeit with the consensus on the role of LCR region in regulating viral gene expression [29, 30], it remains undetermined which methylation patterns, hypomethylation or hypermethylation, in this region are associated with the risk of cervical cancer [25, 27, 31, 32]. Our current study showed that high methylations in CpG 31, 37, or 43 were associated with CIN3+ risk, which were consistent with Sun’s finding [27], but different from Fertey’s finding s[32]. These discrepancies among these observations may partly result from study design (e.g., prospective cohort, nested case-control, or cross-sectional study), methylation assays (e.g., pyrosequencing or next-generation bisulfite sequencing), study population, and/or disease endpoints (e.g., CIN2+ versus CIN3 in our study).

Expression of E6 is integral to hrHPV-induced malignant transformation [33]. Our previous studies had demonstrated the feasibility of HPV E6 oncoprotein detection in risk stratification and long-term risk prediction in cervical cancer screening [23, 34]. This present study showed that HPV 16 E6 oncoprotein had the specificity of 86.5% for the detection of 10-year accumulative CIN3+ among HPV 16 positive women although with suboptimal sensitivity of 57.1%. Moreover, we found that the expression of E6 oncoprotein had positive correlation with HPV 16 hypermethylation sites numbers, indicating that different viral events may be involved in the oncogenic transformation.

From the perspective of triaging performances for cumulative CIN3+, methylation panes alone obtained competitive AUCs values compared with that of cytology, which were in line with the results of several other methylation studies with AUCs for the detection of CIN3+ [10, 35]. A methylation pane alone may obtain the sensitivity of 85.7% and the specificity of 78.4% for the dectection of accumulative CIN3+. Meanwhile, combined with E6 oncoprotein, the sensitivity may be improved for this methylation panel albeit with no apparent increased AUC, which indicated that co-testing at least may provide an option for “trade-off” between sensitivity and specificity.

The extension of triaging potentials of HPV 16 methylation to other genotypes is of interest and of practical value. Given that HPV DNA methylation of any genotype is a general phenomenon marking the transition from HPV infection to precancers for all carcinogenic types [18, 36–39], introducing additional HPV genotypes to the methylation panel testing is anticipated to better the performance of triaging HPV positive women. However, weighing the “trade-off” between the sensitivity and specificity is another issue worthy of careful consideration [35]. Clarke et al.’s recent findings also showed that the improved sensitivity from 60.0 to 80.0% obtained by extending five carcinogenic HPV genotypes to twelve carcinogenic genotypes into the methylation triage panels would be at cost of the reduced risk in positives (positive predictive value) from 30.8 to 18.9% [18]. Several studies on the metylation assay using S5 classifiers that consisted of  methylation status of four carcinogenic HPV types 16, 18, 31, and 33, and EPB41L3 also indicated this trend, although the “trade-off” between the sensitivity and specificity could be approached by adjusting the predefined cutoff [35, 40, 41]. More researches or clinical trials might be desirable to determine the optimal genotypes in HPV methylation panels.

Our present study has advantages in using a 10-year prospective cohort to evaluate the association between HPV methylation and E6 oncoprotein and compare their triage performances for the detection of CIN3+ with other triage options such as cytology. In addition, we used CIN3+, a histologically confirmed immediate precancer, as a primary disease endpoint, which might be more reproducible and of more clinical significance for cervical screening than CIN2+ [42]. However, we also acknowledged that an important limitation with our study was the small size of HPV 16 positive women. Although there were two thousands of participants at the baseline study, because the infection rate of HPV 16 was low to less than 5% among asymptomatic female population, the low statistical power due to a small sample size led to wider confidence intervals for the output measurements, e.g., the cumulative risk of CIN3+ and the sensitivity and specificity. For the same reason, we did not differentiate between HPV 16 single infection and HPV 16 coinfections with other types when we analyzed the methylation status; therefore, the risk stratification ability of HPV 16 methylation may be underestimated. In addition, we only measured the methylation levels at baseline, thus were unable to observe the dynamic variations over time at an individual level, and missed the possibility of interpreting the role of continuous hypermethylation of HPV in the development of cervical precancer and cancer. Another limitation was that we only tested the methylation of HPV 16 positive samples, but not for HPV 16 negative women, so CIN3+ in HPV 16 negative women might be missed [43]. Nevertheless, we found that most CIN3+ cases in HPV 16 negative group was positive for any of other high-risk HPV genotypes at baseline. Therefore, integrating multi-type HPV methylation in cervical cancer screening would be helpful to reduce the loss of CIN3+ cases to some extent.

In conclusion, we provided the population-based data for HPV 16 viral methylation and E6 oncoprotein as two novel and promising tests that might be useful as triage tests for HPV16-positive women. We also confirmed the virtues of standalone DNA methylation and E6 oncoprotein of HPV 16 or their combination in prediction of the future risk at cervical precancers and cancer. Extension of carcinogenic type bases on these two tests is expected to have a more attractive clinical value, but the study of large-scale screening population in real-world settings in addition to technical refinement of molecular approaches would be warranted. In an era of HPV primary screening, such translation of these objective and molecular approach tools will no doubt improve the screening algorithm and accelerate the access to cervical cancer screening.

## Materials and methods

### Study population

A total of 1997 non-pregnant 35–45 years old women with no history of screening or hysterectomies were enrolled in Shanxi Province of Cervical Cancer Screening I (SPOCCS I) study in 1999 [44]. All women with intact cervix were eligible for the subsequent three follow-up studies in 2005, 2010, and 2014, respectively [22, 45–47], as illustrated by Fig. 1. In this present study, we took a total of 77 HPV 16 positive women in 2005 as analytic cohort (AC) baseline due to lack of samples collected in 1999 for HPV methylation and E6 oncoprotein detection.

### Gynecological examinations

Participants underwent liquid-based cytology (LBC, Hologic, Massachusetts), Hybrid Capture 2 (HC2) (Qiagen, Germany), visual inspection with acetic acid (VIA) in 1999, 2005, 2010, and 2014 (except for VIA in 2014) [22, 48]. Women with any tests positive result were referred for colposcopy. Biopsy was taken from the locations of suspected cervical abnormalities. Cytological findings were interpreted according to the Bethesda 2001 classification system. Histological findings were categorized according to cervical intraepithelial neoplasia (CIN) classification system. Women with histologically confirmed CIN grade 2 or worse (CIN2+) lesions were recommended for immediate and standard therapy.

### HC2 testing and viral load determination

HC2 assay was conducted to detect the presence of a total of thirteen hrHPV genotypes (type 16/18/31/33/ 35/39/45/51/52/56/58/59/68). Samples were deemed as HPV-positive if the signal strength in relative light units (RLU) compared with standard positive control (RLU/CO) was 1.0 (1 pg/mL, approximately 5000 viral copies) or higher. The semi-quantitative viral loads of women positive for HPV were then categorized into two levels: low viral load (1.0–99.9 RLU/CO) and high viral load (≥ 100.0 RLU/CO), as previously described .

### DNA extraction and HPV genotyping

Total DNA was isolated from 200 μl ThinPrep PreservCyt Solution (Hologic, USA) with cytological exfoliated cervical cell specimens collected in 2005, 2010**,** and 2014 using Total Nucleic Acid Isolation kit (Qiagen, Germany). We measured DNA purity and concentration using a NanoDrop spectrophotometer (Thermo, USA). Then PCR-based reverse hybridization line probe assay (INNO-LiPA Extra) (Innogenetics, Belgium) with a SPF_10_ primers set (SPF_10_-LiPA, DDL diagnostic laboratory, Netherlands) was performed in HC2-positive specimens during 2005**–**2014 to discriminate twenty-eight individual genotypes [47].

### Bisulfite modification and pyrosequencing for quantitative methylation

Two hundred nanograms of DNA from HPV16 positive samples in 2005 were modified using the EpiTect DNA bisulfite conversion kit (Qiagen, Germany). With bisulfite conversion, unmethylated cytosine (C) residues are preferentially deaminated and converted into uracil (U), which is substituted for thymine (T) residues with methylated C’s remaining unmodified during PCR amplification. PCR with 1.5–2 μl bisulfite-converted methylated template DNA in a final 25 μl reaction volume was performed using the Qiagen PyroMark PCR kit with primers sets specified for each CpG site of L1 and long control region (LCR) gene of HPV 16 (Supplementary Table 3). PCR amplification conditions for each primer set were optimized for ideal annealing and extension temperature (ranged from 54 to 60 °C). Pyrosequencing of 10 μl PCR product was followed via PyroMark Q96 ID platform (Qiagen, Germany). The site-specific quantification of methylation was analyzed blindly with respect to disease outcomes. Methylation levels were calculated by the ratio of “C/(C+T)” indicating the proportion of methylated cytosine at each individual CpG site.

### HPV 16 E6 oncoprotein testing

The OncoE6™ Cervical Test (Arbor Vita Corporation) is an immunochromatographic test using lateral flow format to detect the E6 oncoproteins of HPV type 16, 18, and 45 (only HPV 16 was taken into consideration in this study), as previously described [22]. Briefly, the test result is visually determined by inspection of a red line indicating the presence of the HPV 16 E6 oncoproteins. All HC2 positive cytological samples collected in 2005 and 2014 were tested for HPV 16 E6 oncoproteins, including HPV16 positive specimen in 2005 and 2014 in this study. Specimens tested as hrHPV negative were regarded as HPV 16 E6 oncoprotein negative.

### Statistical analysis

The study in 2005 was taken as the baseline of this analytic cohort (AC) study. Histologically confirmed CIN3+ was taken as primary study endpoint. With the methylation data from HPV 16 positive specimens in 2005, a total of 30 CpG sites of HPV 16 L1 and LCR region were included in the final analysis after exclusion of CpG sites due to the invalid data (Supplementary figure 1). The site-specific methylation levels were described with median and quartile values, and further dichotomized reference into high or low methylation was the third quartile (75%) of the distribution for that site in the AC baseline women without CIN2+.

The 5-year/10-year cumulative incidence rate (CIR) of CIN3+ or CIN2+ during up to ten years of follow up among HPV 16 positive women without CIN2+ stratified by baseline site-specific methylation status (high/low methylation) was estimated by Kaplan-Meier methods and compared by log-rank test. Cox proportional hazard models were used to identify significant high-methylation CpG sites. The 5-year/10-year accumulative risk of CIN3+ or CIN2+ associated with the number of high-methylation sites at baseline was evaluated as site-specific methylation status. We calculated E6 positivity rate with respect to the baseline numbers of high-methylation sites, cross-sectional in 2005, prospective in 2014, and accumulative either in 2005 or in 2014, respectively. Similarly, we calculated HPV 16 positivity frequency with respect to the baseline numbers of high-methylation sites. As three tests for genotyping were conducted during 2005–2014 among baseline HPV 16 positive women, HPV16 infection frequency could be once, twice, or three times.

Methylation panels were further explored based on the number of high-methylation CpG sites with statistical significance in Cox proportional hazard analysis of 10-year risk at CIN3+. Two panels consisted of the same CpG sites but with different number of high-methylation sites as cutoff value. Panel A was regarded as positive if two or more out of these significant CpG sites were hypermethylated, and panel B used at least three hypermethylated CpG sites as cutoff. We combined methylation panel and E6 oncoprotein with the definition of positivity for the condition if either test was positive. We compared 10-year CIRs of CIN3+ and hazard ratio by methylation status and E6 oncoprotein status standalone or their combinations with those by other triage approaches including cytology, viral load**,** and VIA.

Finally, we evaluated the clinical performance of triaging HPV 16 positive women for all triage methods in terms of colposcopy referral rate, sensitivity, specificity, positive predictive value, negative predictive value, and area under curve (AUC) for 10-year cumulative incident CIN3+. Paired Chi-square test was used for comparison of sensitivity and specificity and Z tests for AUC values. All statistical tests were two tailed with a 0.05 significance level. All analyses were performed the using SAS 9.2 and R3.1 software.

## Supplementary information

**Additional file 1: Figure S1.** Site-specific methylation levels of HPV 16 by disease outcomes

**Additional file 2: Figure S2.** Five-year cumulative incidence rate and hazard ratio of CIN3+ in HPV 16 positive women with site-specific high methylation of HPV 16

**Additional file 3: Figure S3.** Five-year cumulative incidence rate and hazard ratio of CIN2+ in HPV 16 positive women with site-specific high methylation of HPV 16

**Additional file 4: Figure S4.** Ten-year cumulative incidence rate and hazard ratio of CIN2+ in HPV 16 positive women with site-specific high methylation of HPV 16

**Additional file 5: Table S1.** Cumulative incident risk of cervical lesions by the numbers of positive HPV 16 methylation sites

**Additional file 6: Table S2.** Methylation status of six significant CpG site of  HPV 16  in 2005 in all accumative incident CIN3+ women

**Additional file 7: Table S3.** Primers for PCR and pyrosequencing of methylation at each CpG site of HPV 16 L1 and LCR genes

## Data Availability

All data generated or analyzed during this study are included in this published article.

## Abbreviations

HPV: Human papillomavirus
L1: Late gene 1
LCR: Long control region
HC2: Hybrid Capture 2
CIR: Cumulative incidence rate
LBC: Liquid-based cytology
VIA: Visual inspection with acetic acid
CIN: Cervical intraepithelial neoplasia
CIN2+: CIN grade 2 or worse
CIN3+: CIN grade 3 or worse
AC: Analytic cohort
FU: Follow up
