# Supplementary figures and images for: Risk stratification of HPV 16 DNA methylation combined with E6 oncoprotein in cervical cancer screening: a 10-year prospective cohort study
Source: Clin Epigenetics. 2020 May 7;12:62. doi: 10.1186/s13148-020-00853-1 (PMC7204324; doi:10.1186/s13148-020-00853-1)

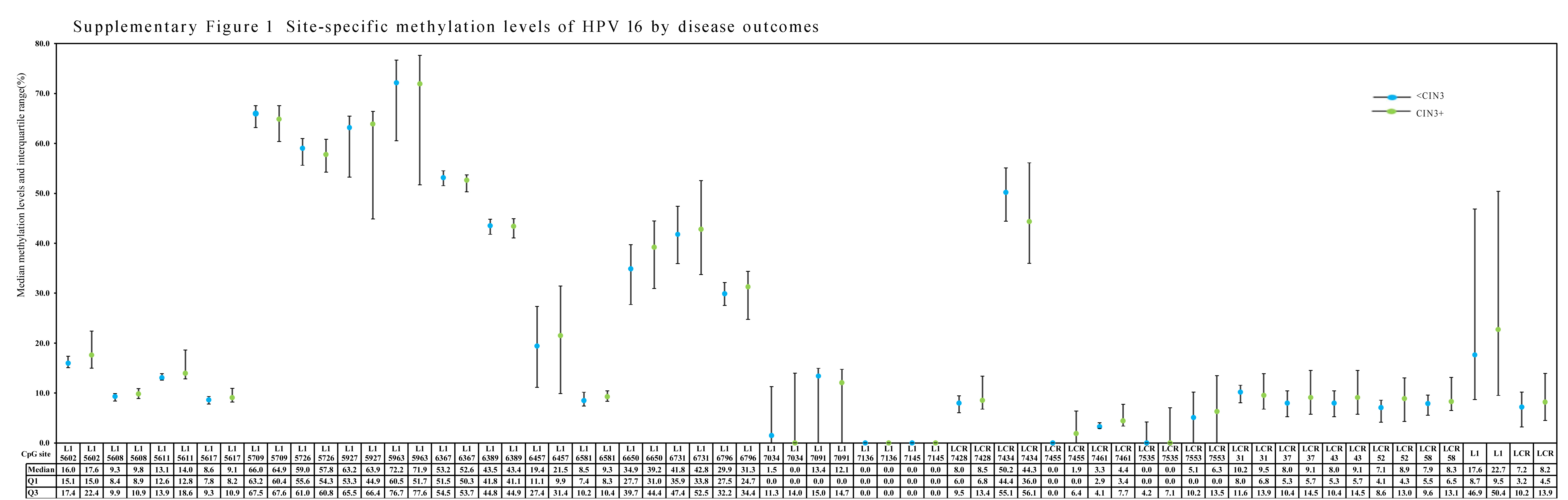

Supplement: Supplementary file 1 — Additional file 1: Figure S1. Site-specific methylation levels of HPV 16 by disease outcomes [file 13148_2020_853_MOESM1_ESM.jpg]

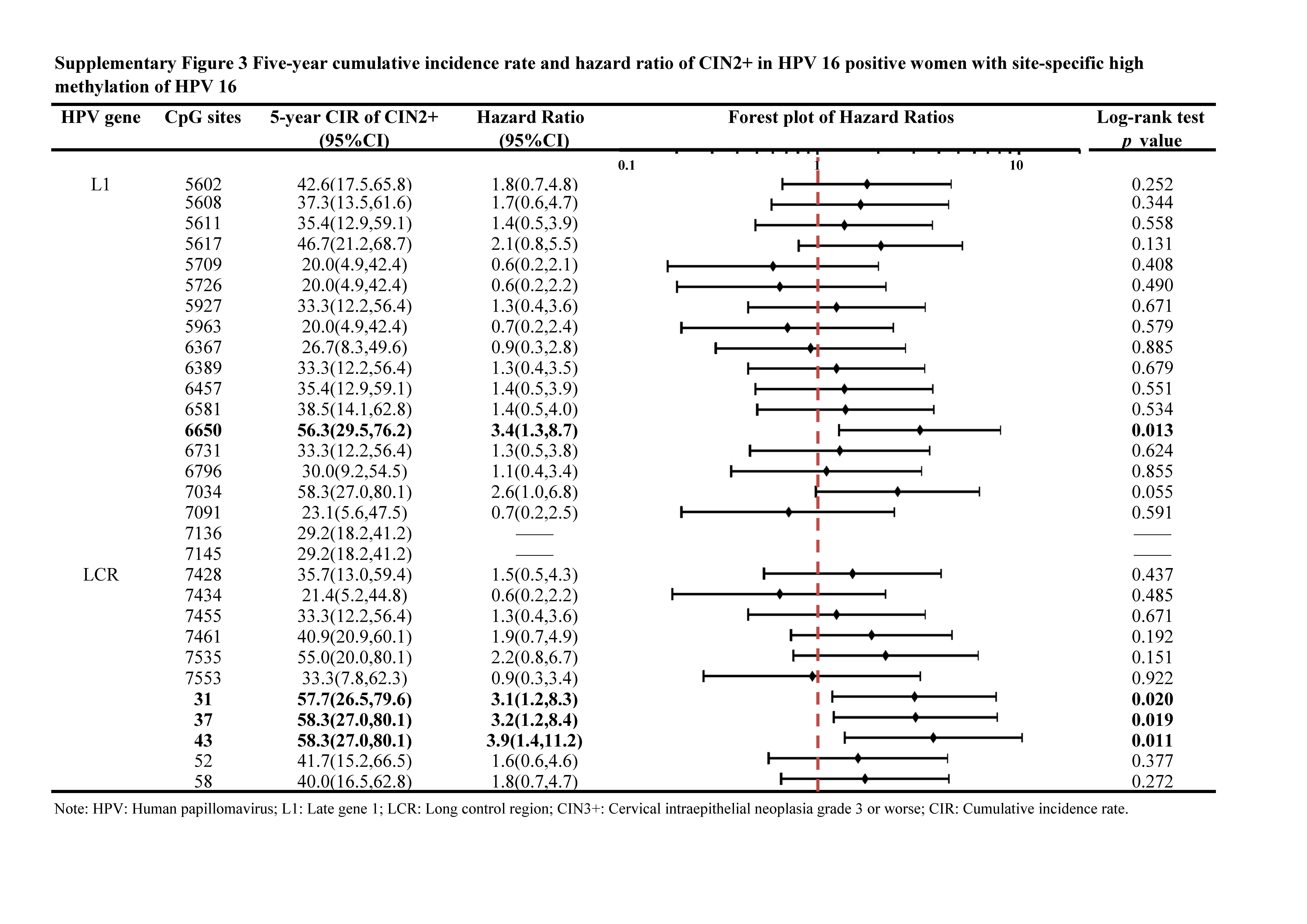

Supplement: Supplementary file 3 — Additional file 3: Figure S3. Five-year cumulative incidence rate and hazard ratio of CIN2+ in HPV 16 positive women with site-specific high methylation of HPV 16 [file 13148_2020_853_MOESM3_ESM.jpg]

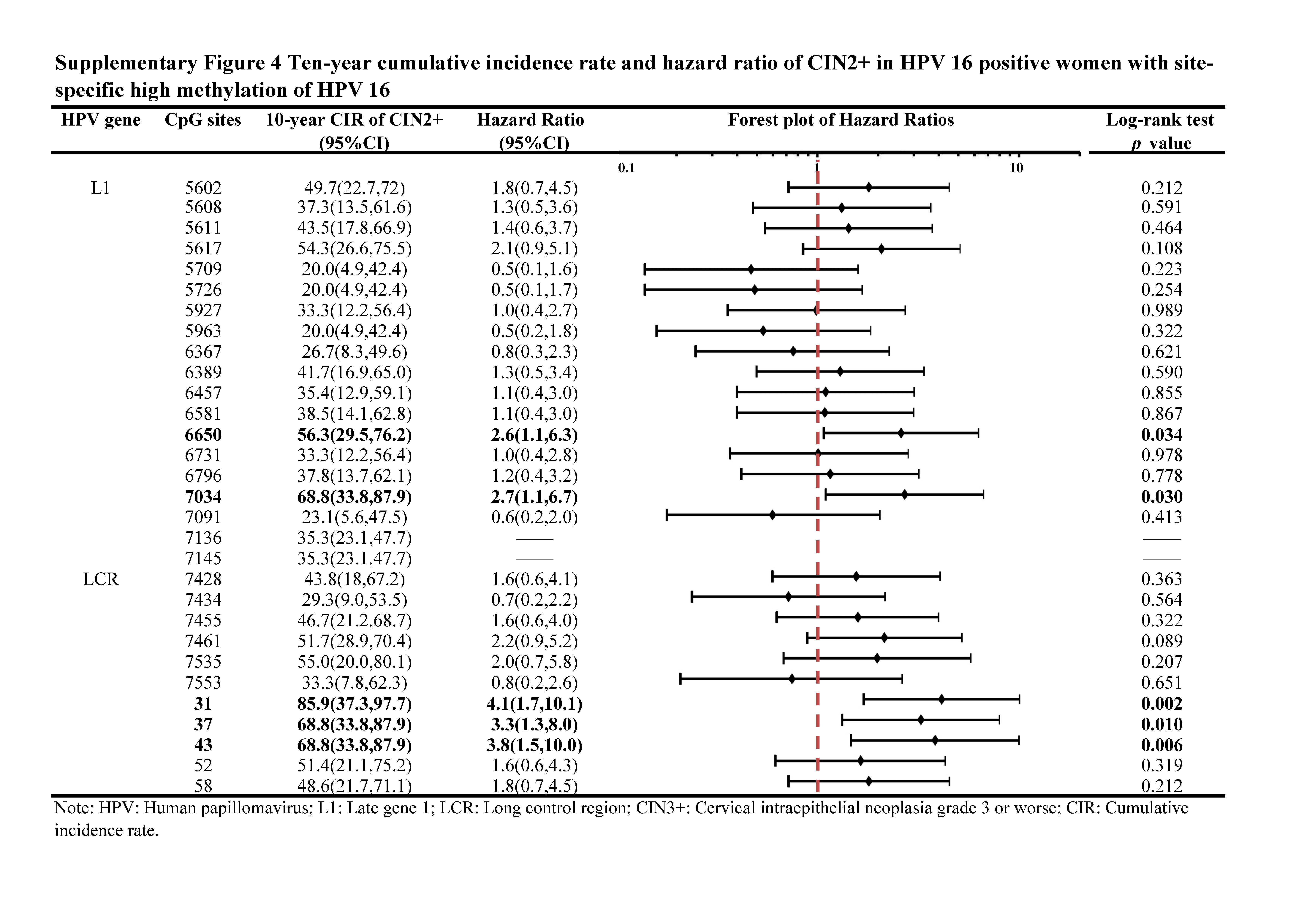

Supplement: Supplementary file 4 — Additional file 4: Figure S4. Ten-year cumulative incidence rate and hazard ratio of CIN2+ in HPV 16 positive women with site-specific high methylation of HPV 16 [file 13148_2020_853_MOESM4_ESM.jpg]
