# Supplementary material for: Risk stratification of HPV 16 DNA methylation combined with E6 oncoprotein in cervical cancer screening: a 10-year prospective cohort study
Source: Clin Epigenetics. 2020 May 7;12:62. doi: 10.1186/s13148-020-00853-1 (PMC7204324; doi:10.1186/s13148-020-00853-1)
Supplement: Supplementary file 5 — Additional file 5: Table S1. Cumulative incident risk of cervical lesions by the numbers of positive HPV 16 methylation sites [file 13148_2020_853_MOESM5_ESM.doc]

Supplementary table 1 Cumulative incident risk of cervical lesions by the numbers of high-methylation HPV 16 methylation sites

| Numbers of HPV 16 CpG sites* | | No. individual | 5-year cumulative CIN2+ | | |  | 5-year cumulative CIN3+ | | | | 10-year cumulative CIN2+ | | |
| --- | --- | --- | --- | --- | --- | --- | --- | --- | --- | --- | --- | --- | --- |
|  |  |  | Incidence | HR **(**95%CI) | *p*^#^ |  | Incidence | HR **(**95%CI) | *p*^#^ |  | Incidence | HR **(**95%CI) | *p*^#^ |
| L1 | 0 | 26 | 11.5(4.0,29.0) | 1(1(Ref.).) | 0.001 |  | 7.7(2.1,24.1) | 1(Ref.) | <0.001 |  | 15.4(6.2,33.5) | 1(Ref.) | 0.002 |
|  | 1 | 28 | 28.6(15.3,47.1) | 3.0(0.8,11.2) |  |  | 10.7(3.7,27.2) | 1.7(0.3,10.0) |  |  | 35.7(20.7,54.2) | 2.8(0.9,8.9) |  |
|  | 2-3 | 8 | 75.0(40.9,92.9) | 7.4(1.8,29.5) |  |  | 75.0(40.9,92.9) | 11.0(2.2,54.6) |  |  | 75.0(40.9,92.9) | 6.9(1.9,25.1) |  |
| LCR | 0 | 29 | 17.2(7.6,34.6) | 1(Ref.) | 0.014 |  | 6.9(1.9,22.0) | 1(Ref.) | 0.003 |  | 17.2(7.6,34.6) | 1(Ref.) | 0.003 |
|  | 1 | 21 | 23.8(10.6,45.1) | 1.5(0.4,5.1) |  |  | 14.3(5.0,34.6) | 2.2(0.4,13.2) |  |  | 33.3(17.2,54.6) | 2.2(0.7,6.9) |  |
|  | 2-3 | 12 | 58.3(32.0,80.7) | 4.3(1.4,13.5) |  |  | 50.0(25.4,74.6) | 9.1(1.8,45.1) |  |  | 66.7(39.6,86.2) | 5.8(1.9,17.8) |  |
| L1+LCR | 0 | 14 | 7.1(1.3,31.5) | 1(Ref.) | <0.001 |  | 0(0.21,5.0) | - |  |  | 7.1(1.3,31.5) | 1(Ref.) | <0.001 |
|  | 1 | 22 | 18.2(7.3,38.5) | 2.7(0.3,23.8) |  |  | 9.1(2.5,27.8) | 1(Ref.) | <0.001 |  | 22.7(10.1,43.4) | 3.4(0.4,29.3) |  |
|  | 2 | 14 | 28.6(11.7,54.7) | 4.6(0.5,41.4) |  |  | 14.3(4.0,39.9) | 1.7(0.2,12.3) |  |  | 35.7(16.4,61.2) | 6.2(0.7,53.4) |  |
|  | 3-6 | 12 | 66.7(39.1,86.2) | 12.2(1.5,97.8) |  |  | 58.3(32.0,80.7) | 8.0(1.7,38.4) |  |  | 75.0(46.8,91.1) | 17.7(2.2,141.2) |  |

*Six statistically CpG sites of HPV 16 are included: CpG 5602, 6650 and 7034 in L1 gene and 7461, 31 and 37 in LCR gene, respectively.

# *p* value for log-rank **(**Mantel-Cox) test.

Abbreviations: HPV: Human papillomavirus; L1= Late gene 1; LCR: Long control region; CIN2+: Cervical intraepithelial neoplasia **(**CIN) grade 2 or worse **(**CIN2+); CIN3+: Cervical intraepithelial neoplasia **(**CIN) grade 3 or worse **(**CIN3+); HR: Hazard ratio; CI: Confidence interval.
