# Supplementary material for: Risk stratification of HPV 16 DNA methylation combined with E6 oncoprotein in cervical cancer screening: a 10-year prospective cohort study
Source: Clin Epigenetics. 2020 May 7;12:62. doi: 10.1186/s13148-020-00853-1 (PMC7204324; doi:10.1186/s13148-020-00853-1)
Supplement: Supplementary file 6 — Additional file 6: Table S2. Methylation status of six significant CpG site of HPV 16 in 2005 in all accumative incident CIN3+ women [file 13148_2020_853_MOESM6_ESM.docx]

| No. | Methylation status for six significant CpG sites in 2005 | | | | | | |  | Disease outcomes | | |
| --- | --- | --- | --- | --- | --- | --- | --- | --- | --- | --- | --- |
|  | 5602 | 6650 | 7034 | 7461 | 31 | 37 | Numbers* |  | in 2005 | in 2010 | in 2014 |
| 1 | Low | High | Low | High | High | High | 4 |  | Normal | CIN3 | —— |
| 2 | Low | Low | Low | High | Low | Low | 1 |  | Normal | CIN3 | —— |
| 3 | Low | High | Low | Low | Low | Low | 1 |  | CIN2 | CIN3 | —— |
| 4 | Low | Low | Low | Low | High | High | 2 |  | Normal | CIN3 | —— |
| 5 | High | Low | High | Low | Low | Low | 2 |  | CIN3 | CIN3 | —— |
| 6 | Low | Low | High | High | High | High | 4 |  | CIN1 | Normal | CIN3 |
| 7 | High | Low | High | Low | Low | Low | 2 |  | Normal | CIN3 | CIN3 |
| 8 | Low | High | High | High | Low | Low | 3 |  | CIN1 | CIN3 | CIN3 |
| 9 | High | Low | Low | Low | Low | Low | 1 |  | Normal | CIN3 | CIN2 |
| 10 | Low | High | High | High | Low | Low | 3 |  | CIN3 | CIN3 | —— |
| 11 | High | High | High | High | High | High | 6 |  | CIN1 | CIN3 | CIN3 |
| 12 | High | High | Low | High | Low | Low | 3 |  | CIN3 | —— | —— |
| 13 | Low | High | Low | Low | High | High | 3 |  | CIN1 | CIN3 | —— |
| 14 | Low | Low | Low | Low | Low | Low | 0 |  | CIN3 | —— | —— |
| 15 | High | Low | Low | High | Low | Low | 2 |  | Normal | Normal | CIN3 |
| 16 | High | High | Low | High | Low | Low | 3 |  | Normal | CIN3 | CIN3 |
| 17 | Low | Low | High | High | Low | Low | 2 |  | CIN2 | CIN3 | CIN3 |
| 18 | Low | Low | High | High | Low | Low | 2 |  | Normal | CIN2 | CIN3 |
| 19 | High | High | High | High | High | High | 6 |  | CIN1 | SCC | —— |
| 20 | High | High | Low | High | High | High | 5 |  | Normal | SCC | —— |

Supplementary Table 2 Methylation status of six CpG sites of HPV 16 in 2005 in all accumulative CIN3+ women

* Number of CpG sites with high-methylation

CIN1: Cervical intraepithelial neoplasia grade 1; CIN2: Cervical intraepithelial neoplasia grade 2; CIN3: Cervical intraepithelial neoplasia grade 3; SCC: squamous cell carcinoma; CIN3+: Cervical intraepithelial neoplasia grade 3 or worse **(**CIN3+)
