# Supplementary material for: Risk stratification of HPV 16 DNA methylation combined with E6 oncoprotein in cervical cancer screening: a 10-year prospective cohort study
Source: Clin Epigenetics. 2020 May 7;12:62. doi: 10.1186/s13148-020-00853-1 (PMC7204324; doi:10.1186/s13148-020-00853-1)
Supplement: Supplementary file 7 — Additional file 7: Table S3. Primers for PCR and pyrosequencing of methylation at each CpG site of HPV 16 L1 and LCR genes [file 13148_2020_853_MOESM7_ESM.docx]

**Supplementary table 3 Primers for PCR and pyrosequencing of methylation at each CpG site of HPV 16 L1 and LCR genes**

| **_Genes_** | **_CpG sites_** | **_Targeting sequences_** | **_Primers for PCR and pyrosequencing_** | **_PCR production (bp)_** | **_Annealing（Tm℃）_** |
| --- | --- | --- | --- | --- | --- |
| _L1_ | _5602 5608 5611 5617_ | _TTAYGAAAAY GAYGTAAAYG TTTATTATAT TTTTTTTTAG ATGT_ | _F1: AGGGTTTTTATAATATATAATTATTGTTGA_  _R1: AAACAAATAAACAATAACCTCACTAAAC_  _S1: TTATTTATATTTTAGTTATTATATG_ | _149_ | _46_ |
| _L1_ | _5709 5726_ | _TTGTAAGTAY GGATGAATAT GTTGTAYGTA TAAATATATA TTATTATGTA GG_ | _F1: TTTTGGTTGTTTAGTGAGGTTATTG_  _R1: ATATCCAACTACAAATAATCTAAATATTCC_  _S1：TGTTTTTTGTTTTAGTATTTAAGG_ | _135_ | _48_ |
| _L1_ | _5927 5963_ | _TATATAGYGG TTGGTTTGGG TTTGTGTAGG TGTTGAGGTA GGTYGTGGTT AGTTATTAGG TGTGGGTAT_ | _F1：TTGATTTTAATAAGTTTGGTTTTTTTGATA_  _R1: AATAACCACTAATACCCACACCTAAT_  _S1: TTGATATTTTATTTTATAATTTAGA_ | _129_ | _46_ |
| _L1_ | _6367 6389_ | _YGATAGTTTA TTTTTTTATT TAYGAAGGGA ATAAATGTTT GTTAGATA_ | _F1: TGATATTGGTTTTGGTGTTATGGATTTTA_  _R1: TTTCACCAACAATACCAACCCTATTA_  _S1：ATGGTGTTAGAATTATATGG_ | _206_ | _52_ |
| _L1_ | _6457_ | _AYGATTTATA TATTAAAGGT TTTGGGT_ | _F1：GTTGGTATTGTTGGTGAAAATGTAT_  _R1：AACTAAATTTACAATAAACCCAAAACCTT_  _S1：TGTTGGTGAAAATGTATTAG_ | _72_ | _50_ |
| _L1_ | _6581_ | _TCRTTATAAC CAATAAAATT TATTAAAT_ | _F1：TAGTGGTTTTATGGTTATTTTTGATGTTTA_  _R1：TACCCCAACAAATACCATTATTATAACCCT_  _S1：AATACCATTATTATAACCCTATAC_ | _95_ | _48_ |
| _L1_ | _6650_ | _TATTATAYGT AGTATAAATA TGTTATTATG TGT_ | _F1：GGTTATAATAATGGTATTTGTTGGGGTAA_  _R1：AACTATAAATCATATTCCTCCCCATATC_  _S1：AATTATTTGTTATTGTTGTTGA_ | _170_ | _52_ |
| _L1_ | _6731_ | _ATTTAYGATA TGGGGAGGAA TATGATTTAT A_ | _F1：GGTTATAATAATGGTATTTGTTGGGGTAA_  _R1：TCCAATCCTCCAAAATAATAAAATTCA_  _S1：ATAAAAATATTAATTTTAAGGAGT_ | _256_ | _50_ |
| _L1_ | _6796_ | _YGTTATGATA TATATATATT TTATGA_ | _F1：GATATGGGGAGGAATATGATTTATAGTT_  _R1：ATTCCAATCCTCCAAAATAATAAAATTCA_  _S1：TGTGTAAAATAATTTTAATTGTAGA_ | _116_ | _50_ |
| _L1_ | _7034_ | _AYGTAAATTT TTATTATAAG TAGGATT_ | _F1：GGAAAAGTTTTTTGTAGATTTAGATTAGTT_  _R1：AATTTTAATTTAACCTTCAATCCTACTTAT_  _S1：AGATTTAGATTAGTTTTTTTTAGG_ | _84_ | _48_ |
| _L1_ | _7091_ | _AAAYGAAAAG TTATATTTAT TATTTTATT_ | _F1：AGTAGGATTGAAGGTTAAATTAAAAT_  _R1：TTATATACAATAAATAACCACAACACAATT_  _S1：GGTTAAATTAAAATTTATATTAGGA_ | _327_ | _50_ |
| _L1_ | _7136 7145_ | _TTAAAYGTAA AAAAYGTAAG TTGTAAGTAT TGTATGTATG_ | _F1：AGTAGGATTGAAGGTTAAATTAAAAT_  _R1：AATTTATATACAATAAATAACCACAACACA_  _S1：ATTATTTTATTTATTTTTATAATTG_ | _330_ | _50_ |
| _LCR_ | _7270_ | _TAYGTGTGTA TGTGTTTTTA AATGTTTG_ | _F1：AGTAGGATTGAAGGTTAAATTAAAAT_  _R1：TTATATACAATAAATAACCACAACACAATT_  _S1：GTGTTTGTATGTATGGTATAATAAA_ | _327_ | _48_ |
| _LCR_ | _7428 7434 7455 7461_ | _AACCRAATTC RATTAAAACT ACAAAATAAC CRCTAACRCT ACAAAATATA ATATATATAA AAC_ | _F1：TGTGTTGTGGTTATTTATTGTATATAAATT_  _R1: AACCATAATTACTAACATAAAACTATTTAA_  _S1：ACATTTTATACCAAAAAACATAC_ | _174_ | _46_ |
| _LCR_ | _7535 7553_ | _TAYGTTTTTT GTTTGTTATG YGTGTTAAAT TTTTGTTTTT TTGATT_ | _F1：TTGGTATAAAATGTGTTTTTTTAAATAGT_  _R1：AAACAAACCAAAAATATATACCTAACAAC_  _S1：TGTTAGTAATTATGGTTTAAATTTG_ | _249_ | _46_ |
| _LCR_ | _7676 7682 7694_ | _TTATGYGTTA AYGTTTTATA TATYGTTGTT AGGTATATAT TTTTGGTTT_ | _F1：GTATGTTTTTTGGTATAAAATGTGTTT_  _R1: ATTAAAACAAACCAAAAATATATACCTAAC_  _S1：TGTGTTATATAAAATAAATTA_ | _262_ | _46_ |
| _LCR_ | _7862_ | _YGTTTTGGGT TATATATTTA TAAGTA_ | _F1：TGTAAAATTGTATATGGGTGTGTGTA_  _R1：CCTATAAATCCTAAAACATTACAATTCTCT_  _S1：ATGGGTGTGTGTAAAT_ | _196_ | _50_ |
| _LCR_ | _31 37 43 52 58_ | _AACCRATTTC RATTCAACCR ATTTCRATTA CRCCCTTAAT TTTATACATA AATTATT_ | _F1：ATTTGTAAAATTGTATATGGGTGTGTG_  _R1：CCTATAAATCCTAAAACATTACAATTCTCT_  _S1：ACATAAAATATCTACTTTTATACT_ | _199_ | _50_ |
